# Access to antibiotics in New Delhi, India: implications for antibiotic policy

**DOI:** 10.1186/2052-3211-6-6

**Published:** 2013-08-12

**Authors:** Anita Kotwani, Kathleen Holloway

**Affiliations:** 1 Department of Pharmacology, V. P. Chest Institute, University of Delhi, Delhi, India; 2 Essential Drugs and Other Medicines, World Health Organization, Regional Office of South East Asia, New Delhi, India

**Keywords:** Access, Antibiotics, Antibiotic availability, Antibiotic price, Primary care, Public health sector, Private retail pharmacies, Procurement price

## Abstract

**Objective:**

The present survey was conducted to investigate the price and availability of a basket of 24 essential antibiotics and eight high-end antibiotics at various levels of health care in public and private sector in National Capital Territory of Delhi, India using standardized WHO/HAI methodology.

**Methods:**

Data on procurement price and availability was collected from three public healthcare providers in the state: the federal (central) government, state government and Municipal Corporation of Delhi (MCD). Overall a total of 83 public facilities, 68 primary care, 10 secondary cares and 5 tertiary care facilities were surveyed. Data was also collected from private retail (n = 40) and chain pharmacies (n = 40) of a leading corporate house. Prices were compared to an international reference price (expressed as median price ratio-MPR).

**Results:**

Public sector: Delhi state government has its essential medicine list (Delhi state EML) and was using Delhi state EML 2007 for procurement; the other two agencies had their own procurement list. All the antibiotics procured including second and third generation antibiotics except for injections were available at primary care facilities. Antibiotic available were on the basis of supply rather than rationality or the Delhi state EML and none was 100% available. There was sub-optimal availability of some essential antibiotics while other non-essential ones were freely available. Availability of antibiotics at tertiary care facilities was also sub-optimal. Private sector: Availability of antibiotics was good. For most of the antibiotics the most expensive and popular trade names were often available. High-end antibiotics, meropenam, gemifloxacin, and moxifloxacin were commonly available. In retail pharmacies some newer generation non-essential antibiotics like gemifloxacin were priced lower than the highest-priced generic of amoxicillin + clavulanic acid, azithromycin, and cefuroxime aexitl.

**Conclusions:**

Inappropriate availability and pricing of newer generation antibiotics, which may currently be bought without prescription, is likely to lead to their over-use and increased resistance. All providers should follow the EML of whichever of the three concerned Delhi public sector agencies that it is under and these EMLs should follow the essential medicine concept. The Indian regulatory authorities need to consider urgently, drug schedules and pricing policies that will curtail inappropriate access to new generation antibiotics.

## Introduction

Antimicrobial resistance (AMR) is rapidly increasing globally, therefore suitable policy and actions are urgently needed to combat AMR [1]. The widely held consensus for the rapid increase in development of AMR is over-prescribing and inappropriate use of antibiotics [2,3]. This consensus leads in turn to the need for having an antibiotic policy that should ensure critical selection and appropriate use of antibiotics [4]. Since health systems and prescribing behavior are varied and complex, a nationally or locally coordinated antibiotic policy, contextualized to the particular country/region, will be needed to have any substantial impact on appropriate antibiotic use.

The first step in combating inappropriate use of antibiotics is to measure access and use of antibiotic in order to quantify the scale of the problem and to have a baseline data to compare and evaluate the impact of any intervention. In a number of developed countries, extensive surveillance programs have been developed to study patterns of antibiotic use and guidelines for antibiotic use [5-7]. However, the ability to undertake such surveillance is lacking in resource-constrained settings. The problem of AMR, appropriate availability and inappropriate use of antibiotics has received relatively little recognition in developing countries [8]. Recently, a few collaborative studies were conducted in India for developing a validated methodology for measuring consumption and antibiotic use in the community and hospital [8-10]. Overprescribing, misuse of antibiotics and low adherence to treatment guidelines were seen in both public and private settings [10-13]. However, there is no literature available in India on access, availability and price of various antibiotics at public and private retail pharmacies. Hence, the present reported survey was undertaken (July-October 2011) with the objective to study to measure the access of various antibiotics in public and private sectors in New Delhi using a standardized methodology of World Health Organization and Health Action International (WHO/HAI) [14]. The survey was undertaken with the ultimate objective to move towards enabling an antibiotic policy that will encourage availability of appropriate antibiotics (and not inappropriate ones) and hence their rational use at different level of healthcare.

## Methods

### State background

Delhi, the capital of India is known as the National Capital Territory of Delhi (NCT Delhi).

Three main public sector health care providers in NCT Delhi are: the Central (federal) Government under the Ministry of Health and Family Welfare (MoH&FW), the Directorate of Health Services (DHS) in the Government of NCT (GNCT) Delhi and another public sector provider in Delhi city – the Municipal Corporation of Delhi (MCD). All citizens can visit and avail the free services in the facilities of the three public healthcare providers. All these public sectors have their own procurement systems and essential or procurement list of medicines.

In the private sector, recently, chain pharmacies have entered the Indian retail sector. Therefore, both types of retail pharmacies were included in the survey, the traditional private retail pharmacies and the chain pharmacies of a leading corporate house.

### Sampling

This survey followed the WHO/HAI methodology [14] and was conducted in all eight districts of Delhi and surveyed five facilities in each district covering the entire Delhi state.

#### ***Public sector***

Central procurement prices were collected from all the three centralized public sector procurement agencies/department of healthcare providers. Tertiary care facilities under central government (CG) also undertake independent medicine procurement to augment the supply from the central government procurement agency. Procurement prices were also collected from these tertiary care facilities; namely, Ram Manohar Lohia (RML) Hospital and Safdurjung Hospital (SH) which have common pooled procurement (labeled as CG1) and Lady Hardinge Medical College (LH) which has independent procurement (labeled as CG2).

Medicines are provided for free in all public facilities hence only antibiotic availability was collected from the various public facilities. For each district one secondary care hospital and four primary health care (dispensaries) were randomly selected. In one of the districts a tertiary care instead of secondary care hospital was enrolled. Overall a total of 83 facilities (40 facilities under GNCT, Delhi, 40 under MCD and 3 tertiary care facilities of CG) were surveyed (Table 1).

**Table 1:** Number of facilities in each survey area under public and private sector

**S.No **	**Survey area**	Public sector*			**Private Sector#**	
	**District**	**National capital territory of Delhi Government**	**Central government**	**Municipal corporation of Delhi**	**Retail pharmacy**	**Retail chain pharmacy**
1	Central	5	2	5	5	5
2	South	5	1	5	5	5
3	East	5		5	5	5
4	North	5		5	5	5
5	North East	5		5	5	5
6	North West	5		5	5	5
7	South West	5		5	5	5
8	West	5		5	5	5
**Total**		40	3	40	40	40
	**Grand total**	**83**			**80**	

*For public sector one version of each medicine, the lowest-priced generic (LPG) were collected for both price and availability as public sector procure only one generic version of each medicine.

#For private sector, Seven core antibiotics were surveyed in three versions, Originator brand (OB), Highest-priced generic (HPG) and Lowest-priced generic (LPG); Seventeen supplementary antibiotics and eight high end antibiotics were surveyed in two versions, HPG and LPG.

#### ***Private sector***

The private sector samples were identified by selecting one retail pharmacy outlet in each sector that is geographically closest to each public outlet. In each district five retail pharmacies and five retail chain pharmacies were included. Thus a total of 80 facilities were surveyed.

### Antibiotics surveyed

The WHO/HAI methodology identifies a basket of 30 core medicines – 14 essential medicines from global burden of diseases and 16 specific for a particular region, South East Asia. Of these 30 medicines, seven are antibiotics in different dosage forms (Table 2). To these core seven antibiotics, a supplementary list of 17 antibiotics from various classes were added as mentioned in Table 3 on the basis of their inclusion in Delhi State essential medicine list (EML). Also, a further eight high-end antibiotics (listed in the footnote of Table 3) were surveyed in secondary care and tertiary care public facilities and private sector pharmacies.

**Table 2:** List of seven antibiotics mentioned in WHO/HAI methodology

**Antibiotic no.**	**Antibiotic name**	**Medicine strength**	**Dosage form**
1	Amoxicillin	500 mg	cap/tab
2	Amoxicillin suspension	25 mg/ml	millilitre
3	Ceftriaxone injection	1 g/vial	vial
4	Ciprofloxacin	500 mg	cap/tab
5	Co-trimoxazole suspension	8 + 40 mg/ml	mililitre
6	Doxycycline	100 mg	cap/tab
7	Gentamicin eye drops	0.3%	millilitre

**Table 3:** List of Seventeen antibiotics added

**Antibiotic no.**	**Antibiotic name**	**Medicine strength**	**Dosage form**
1	Amoxicillin + Clavulanic acid	500 g + 125 mg	cap/tab
2	Amoxicillin 250	250 mg	Tab/cap
3	Amoxicillin + Clavulanic acid Syrup	200 mg + 28.5 mg/5 ml	mililitre
4	Ampicillin Suspension	125 mg/5 ml	mililitre
5	Azithromycin	500 mg	Tab/cap
6	Benzathine Penicillin Powder	2.4MU/vial	vial
7	Cefixime	200 mg	Tab/cap
8	Cefuroxime axetil	250 mg	Tab/cap
9	Cefuroxime Suspension	125 mg/5 ml	mililitre
10	Cephalexin	500 mg	Tab/cap
11	Cephalexin Syrup	250 mg/5 ml	mililitre
12	Erythromycin powder for suspension	125 mg/5 ml	mililitre
13	Erythromycin Stearate	250 mg	Tab/cap
14	Gentamicin injection	40 mg/ml	inj
15	Norfloxacin	400 mg	Tab/cap
16	Ofloxacin	200 mg	Tab/cap
17	Roxithromycin	50 mg	Tab/cap

Additional list of eight high-end antibiotics surveyed in hospitals and private pharmacies.

1. Meropenem 500 mg inj.

2. Imipenem + cilastatin 500 mg + 500 mg inj.

3. Colistin inj 1,000,000 units/vial.

4. Vancomycin 500 mg inj.

5. Ceftazidime 1 g vial.

6. Cefepime 1 g inj.

7. Gemifloxacin 320 mg.

8. Moxifloxacin 400 mg.

As per the WHO/HAI methodology [14] data was collected for both the originator brand, and the lowest-priced generic equivalent found at each medicine outlet. Until 2005, the Indian regulatory system recognized process patents not product patent and therefore all medicines that are manufactured in India are generic versions. However, all products carry a brand (trade) name [15]. Originators brands (OBs) in India do not have any additional recognition as originator brand. Often OBs are not available but the same molecules manufactured by other companies with different trade names, branded generics, are available. Therefore, for this survey, in addition to originator brand and lowest-priced generic (LPG) a third version, highest-priced generic (HPG) was added to find out the price and availability of seven core antibiotics available at each facility. For seventeen supplementary antibiotics and eight high-end antibiotics, two versions, highest-priced and lowest-priced generics were surveyed and found at each private facility (Table 1, 2, and 3).

Since the public sector has only one version of each medicine, only the lowest-priced generic (LPG) were collected for both price and availability.

### Data collection, entry and analysis

Trained data collectors visited enrolled facilities with a standardized form and recorded the price and availability of each antibiotic medicine. For public sector, procurement price was collected from three central agencies - Central procurement agency (CPA) for Delhi state government (GNCT, Delhi); procurement department of MCD; and Medical Stores Organization (MSO), procurement department of CG. Procurement price was also collected from two decentralized sites of central government hospitals – CG1 (RML/SH) and CG2 (LH).

Medicine unit prices were entered into Excel spreadsheets with double entry, auto-checking, and automated analysis feature of the workbook developed by WHO/HAI [14]. To facilitate international comparisons, antibiotic prices are expressed as median price ratio (MPR) [16]. The MPR is the local median unit price of a medicine in comparison with the median unit price found in the Management Sciences for Health (MSH) Price Indicator Guide, 2010 [17].

### Ethical approval

Ethical approval of the study was obtained from Vallabhbhai Patel Chest Institute, University of Delhi, India. Permission for data collection was obtained from Health Department, Directorate Health Services (DHS) of Government of NCT Delhi, Municipal Corporation of Delhi, and from Ministry of Health & Family Welfare, Government of India.

## Results

### Public sector

#### ***Procurement prices***

The median MPR of each antibiotic surveyed, whether it is on the Delhi state EML and which procurement agencies procured these antibiotics are shown in Table 4. The 2007 Delhi sate EML was used for purchase of medicines for Delhi state run facilities at the time of the survey in 2011 [18]. Out of 24 antibiotics surveyed, 20 antibiotics were on Delhi state EML used by the CPA, the GNCT, Delhi procurement agency, which was procuring all antibiotics on the list except benzathine penicillin. The other two public sector procurement agencies did not have their own EML but had their list of medicines for procurement prepared by their institute committee.

**Table 4:** Median price ratios (MPRs) of surveyed antibiotics in various public sector procurement agencies in Delhi

**Antibiotic no.**	**Medicine name**	**Delhi state EML 2007**	**Median MPR**	**Procurement agencies**
1	Amoxicillin + Clavulanic acid 500 mg + 125 mg cap/tab	No	0.91	MCD, CG1,CG2
2	Amoxicillin 500 mg cap/tab	Yes	1.22	MCD, CG2,CPA
3	Amoxicillin 250 mg cap/tab	Yes	1.11	MCD, CG1,CG2, CPA
4	Amoxicillin suspension 25 mg/ml	Yes	1.04	MCD,CG1,CG2,CPA
5	Amoxicillin + Clavulanic acid Syrup 200 mg + 28.5 mg/5 ml	No	*	MSO,MCD
6	Ampicillin Suspension 125 mg/5 ml	Yes	0.97	CPA
7	Azithromycin 500 mg cap/tab	No	0.53	MCD,CG2
8	Benzathine Penicillin Powder 2.4MU/vial	Yes	0.48	CG1
9	Cefixime 200 mg cap/tab	No	0.54	MCD
10	Ceftriaxone injection 1 g/vial	Yes	0.50	MSO, MCD, CG1,CG2, CPA
11	Cefuroxime axetil 250 mg cap/tab	Yes	0.64	MSO,MCD, CPA
12	Cefuroxime Suspension 125 mg/5 ml	Yes	0.43	CPA
13	Cephalexin 500 mg cap/tab	Yes	0.83	MCD,CG1,CPA
14	Cephalexin Syrup 250 mg/5 ml	Yes	0.68	MSO,CG2,CPA
15	Ciprofloxacin 500 mg cap/tab	Yes	0.88	MCD,CG1,CG2,CPA
16	Co-trimoxazole suspension 8 + 40 mg/ml	Yes	0.76	MCD,CG1,CG2,CPA
17	Doxycycline 100 mg cap/tab	Yes	1.11	MCD,CG2,CPA
18	Erythromycin powder for suspension 125 mg/5 ml	Yes	0.67	CG1,CG2,CPA
19	Erythromycin Stearate 250 mg cap/tab	Yes	1.03	MSO,MCD,CG1,CG2,CPA
20	Gentamicin eye drops 0.3%	Yes	0.44	MCD,CG1,CG2,CPA
21	Gentamicin injection 40 mg/ml	Yes	0.77	MCD,CG1,CG2,CPA
22	Norfloxacin 400 mg cap/tab	Yes	0.75	MCD,CG1,CG2,CPA
23	Ofloxacin 200 mg cap/tab	Yes	0.59	MSO,MCD,CG1,CG2,CPA
24	Roxithromycin 50 mg cap/tab	Yes	*	MSO,MCD,CPA

*MSO* Medical store organization, the procurement agency for central government hospitals, *MCD* Municipal corporation of Delhi, *CG1* decentralized procurement done by two tertiary care hospitals of central government, *CG2* decentralized procurement done by single tertiary care hospital of central government, *CPA* the procurement agency for Government of National Capital Territory of Delhi.

* MSH price is not available therefore MRP can not be calculated.

A few antibiotics, e.g., azithromycin, ceftriaxone injection, erythromycin, gentamicin eye drops, gentamicin injection, and roxithromycin were found to have large variation in procurement price by different agencies (Table 5). Usually the highest procurement price for these medicines was for the tertiary care facilities (CG2 and CG1) doing independent procurement and the least price was for CPA-GNCT, Delhi which manages pooled procurement for all the facilities under the Delhi state government.

**Table 5:** Variation in unit procurement price in local currency (INR) of few antibiotics by different procurement agencies in New Delhi

**Antibiotic name**	**MSO(central government**	**MCD**	**CG1**	**CG2**	**CPA(Delhi state government)**
Azithromycin cap/tab	No rate available	6.31	No rate available	9.45	No rate available
Ceftriaxone injection	14.99	18.28	16.46	15.50	8.50
Erythromycin Stearate	1.43	1.32	2.04	1.68	0.85
cap/tab					
Gentamicin eye drops	No rate available	0.61	0.81	1.21	0.58
Gentamicin injection	No rate available	1.40	1.53	2.49	1.52
Roxithromycin cap/tab	1.04	1.29	No rate available	No rate available	0.74

*MSO* Medical store organization, the procurement agency for central government hospitals, *MCD* Municipal corporation of Delhi, *CG1* decentralized procurement done by two tertiary care hospitals of central government, *CG2* decentralized procurement done by single tertiary care hospital of central government, *CPA* the procurement agency for Government of National Capital Territory of Delhi, *INR* Indian Rupees, Local currency.

#### ***Availability of surveyed antibiotics***

Table 6 depicts the availability of surveyed antibiotics in primary care (dispensaries), secondary care hospitals and tertiary care level of hospital under Delhi state government, (GNCT, Delhi) and under Municipal Corporation of Delhi (MCD). Three antibiotics which were not in Delhi State EML were found: amoxicillin + clavulanic acid tablet was available at all three levels of health care of Delhi State and MCD and 2 out of three tertiary care facilities of Central Government (CG). Amoxicillin + clavulanic acid syrup and cefixime was available at primary and secondary care facilities of GNCT, Delhi and MCD and only at one tertiary care facility of MCD.

**Table 6:** Availability of surveyed antibiotics in primary, secondary and tertiary care facilities of two public sectors in Delhi, India

**Antibiotic name**	**GNCT Delhi**			**MCD**		
	**Pr. care**	**Second care**	**Tertiary care**	**Pr. care**	**Second care**	**Tertiary care**
	**(n = 32)**	**(n = 7)**	**(n = 1)**	**(n = 36)**	**(n = 3)**	**(n = 1)**
1. amoxicillin 250 + clavulanic acid$	21.9%	42.9%	Available	52.8%	66.7%	Available
2. Amoxicillin 250#	71.9%	57.1%	Available	19.4%	33.3%	N.A
3. Amoxicillin 500#	81.3%	85.7%	Available	30.6%	66.7%	Available
4. Amoxicillin suspension#	62.5%	100.0%	Available	5.6%	0.0%	N.A
5. Amoxicillin + clavulanic acid	31.3%	14.3%	N.A	58.3%	66.7%	Available
Syrup$						
6. Ampicillin suspension#	25.0%	0.0%	N.A	2.8%	0.0%	N.A
7. Azithromycin@	0.0%	14.3%	Available	2.8%	0.0%	N.A
8. Benzathine Penicillin Powder#	0.0%	0.0%	N.A	0.0%	0.0%	N.A
9. Cefixime$	21.9%	14.3%	N.A	25.0%	66.7%	Available
10. Ceftriaxone injection@*	0.0%	57.1%	Available	0.0%	0.0%	N.A
11. Cefuroxime axetil@*	43.8%	71.4%	N.A	47.2%	66.7%	N.A
12. Cefuroxime suspension@*	0.0%	28.6%	N.A	0.0%	0.0%	N.A
13.Cephalexin#*	43.8%	71.4%	N.A	19.4%	33.3%	N.A
14. Cephalexin syrup#*	53.1%	14.3%	Available	8.3%	0.0%	N.A
15. Ciprofloxacin#	37.5%	100.0%	Available	72.2%	100.0%	Available
16. Co-trimoxazole suspension#	56.3%	85.7%	Available	2.8%	0.0%	N.A
17. Doxycycline#	50.0%	71.4%	Available	69.4%	100.0%	Available
18. Erythromycin powder for suspension#	40.6%	42.9%	Available	0.0%	0.0%	N.A
19. Erythromycin stearate#	43.8%	57.1%	Available	0.0%	0.0%	N.A
20. Gentamicin eye drops@	59.4%	71.4%	Available	11.1%	33.3%	N.A
21. Gentamicin injection@	3.1%	28.6%	Available	0.0%	0.0%	N.A
22. Norfloxacin#	84.4%	85.7%	Available	2.8%	0.0%	N.A
23. Ofloxacin@	37.5%	71.4%	Available	47.2%	66.7%	Available
24. Roxithromycin#	40.6%	71.4%	N.A	8.3%	0.0%	N.A

$Not in the Delhi State EML 2007 for Hospitals and dispensaries.

# For primary care (dispensaries) Delhi State EML2007.

#* Reserve antibiotics for restricted use in primary care.

@Antibiotics for hospital; these are in addition to antibiotics for primary care facilities.

* Reserve antibiotics to be used only in case of significant resistance to other antibiotics.

Antibiotics with more than 80% availability at GNCT, Delhi-run primary care were: amoxicillin500 mg and norfloxacin tablets. Antibiotics with more than 80% availability at MCD run primary care were none and at secondary care were ciprofloxacin and doxycycline. The availability of some essential antibiotics e.g. ampicillin suspension, erythromycin suspension and tablets for primary care were sub-optimal, particularly in MCD.

Table 7 shows availability of antibiotics in tertiary care facilities of Central Government. Four antibiotics amoxicillin250 mg, ciprofloxacin, erythromycin stearate, and norfloxacin for OPD patients and two injections ceftriaxone and gentamicin were available in all three tertiary care facilities. By contrast some essential antibiotics e.g. amoxicillin suspension, doxycycline and cephalexin were poorly available.

**Table 7:** Availability of surveyed antibiotics in facilities surveyed in three tertiary care facilities of federal government in Delhi, India

**Medicines**	**CGH(n = 3)**	**LH**	**RML**	**SH**
1. Amoxicillin + clavulanic acid	66.7%	Available	N.A	Available
2. Amoxicillin 250	100.0%	Available	Available	Available
3. Amoxicillin 500	33.3%	Available	N.A	N.A
4. Amoxicillin suspension	33.3%	Available	N.A	N.A
5. Amoxicillin + clavulanic acid Syrup	N.A	N.A	N.A	N.A
6. Ampicillin suspension	N.A	N.A	N.A	N.A
7. Azithromycin	33.3%	Available	N.A	N.A
8. Benzathine Penicillin Powder	66.7%	N.A	Available	Available
9. Cefixime	N.A	N.A	N.A	N.A
10. Ceftriaxone injection	100.0%	Available	Available	Available
11. Cefuroxime axetil	N.A	N.A	N.A	N.A
12. Cefuroxime suspension	N.A	N.A	N.A	N.A
13. Cephalexin	33.3%	N.A	Available	N.A
14. Cephalexin syrup	33.3%	Available	N.A	N.A
15. Ciprofloxacin	100.0%	Available	Available	Available
16. Co-trimoxazole suspension	66.7%	Available	Available	N.A
17. Doxycycline	33.3%	Available	N.A	N.A
18. Erythromycin powder for suspension	66.7%	Available	Available	N.A
19. Erythromycin stearate	100.0%	Available	Available	Available
20. Gentamicin eye drops	66.7%	Available	N.A	Available
21. Gentamicin injection	100.0%	Available	Available	Available
22. Norfloxacin	100.0%	Available	Available	Available
23. Ofloxacin	66.7%	Available	Available	N.A
24. Roxithromycin	N.A	N.A	N.A	N.A

*CGH* Central Government Hospitals.

*LH* Lady Hardinge Medical College & Associated Hospitals.

*RML* Ram Manohar Lohia Hospital.

*SH* Safdarjung Hospital.

### Private sector

#### ***Price-to-patient***

The median MPR for all versions, minimum and maximum MPR found at retail pharmacies and median unit price in local currency is shown in Table 8. Findings were similar at retail chain pharmacies, except for a few antibiotics, like amoxicillin suspension, cephalexin syrup, and gentamicin eye drops whose median MPRs were found to be little less and for few antibiotics, like ciprofloxacin, doxycycline, median MPRs a little more than the median MPRs found at private retail pharmacies.

**Table 8:** Median price ratio, median unit price in local currency and availability of surveyed antibiotics at private retail pharmacies

**No.**	**Antibiotic name**	**Medicine type**	**Median price ratio (MPR)**	**Min**	**Max**	**% of facilities with antibiotic**	**Median unit price in INR**
1	Amoxicillin + Clavulanic acid	Highest Price	4.83	0.48	4.83	95.0%	40.17
	Amoxiciilin + Clavulanic acid	Lowest Price	1.44	0.50	4.83	100.0%	11.99
2	Amoxicillin	Brand	N.A	N.A	N.A	0.0%	N.A
	Amoxicillin	Highest Price	7.45	7.21	7.46	95.0%	9.65
	Amoxicillin	Lowest Price	7.21	5.05	7.45	97.5%	9.33
3	Amoxicillin 250	Highest Price	7.73	7.20	7.73	72.5%	5.90
	Amoxicillin 250	Lowest Price	5.50	4.28	7.73	92.5%	4.20
4	Amoxicillin suspension	Brand	N.A	N.A	N.A	0.0%	N.A
	Amoxicillin suspension	Highest Price	7.02	3.64	7.02	57.5%	1.41
	Amoxicillin suspension	Lowest Price	4.55	3.28	7.04	95.0%	0.91
5	Amoxicillin + Clavulanic acid Syrup	Highest Price	*			62.5%	3.64
	Amoxicillin + Clavulanic acid Syrup	Lowest Price	*			92.5%	1.58
6	Ampicillin Suspension	Highest Price	#			2.5%	
	Ampicillin Suspension	Lowest Price	#			2.5%	
7	Azithromycin	Highest Price	2.06	1.42	2.16	95.0%	30.83
	Azithromycin	Lowest Price	1.57	1.14	2.06	100.0%	23.50
8	Benzathine Penicillin Powder	Highest Price	-	-	-	0.0%	-
	Benzathine Penicillin Powder	Lowest Price	-	-	-	0.0%	-
9	Cefixime	Highest Price	2.53	1.74	2.55	80.0%	19.81
	Cefixime	Lowest Price	1.26	0.87	2.56	97.5%	9.90
10	Ceftriaxone injection	Brand	N.A	N.A	N.A	0.0%	N.A
	Ceftriaxone injection	Highest Price	#			2.5%	
	Ceftriaxone injection	Lowest Price	2.24	1.95	2.24	47.5%	69.00
11	Cefuroxime axetil	Highest Price	5.73	3.59	5.73	55.0%	43.10
	Cefuroxime axetil	Lowest Price	3.23	1.40	5.73	90.0%	24.30
12	Cefuroxime Suspension	Highest Price	#			2.5%	
	Cefuroxime Suspension	Lowest Price	3.16	2.41	3.51	55.0%	4.33
13	Cephalexin	Highest Price	5.62	5.54	5.66	32.5%	16.86
	Cephalexin	Lowest Price	5.40	3.23	5.62	65.0%	16.20
14	Cephalexin Syrup	Highest Price	5.53	4.59	5.53	22.5%	2.27
	Cephalexin Syrup	Lowest Price	4.79	1.55	5.53	50.0%	1.97
15	Ciprofloxacin	Brand	N.A	N.A	N.A	0.0%	N.A
	Ciprofloxacin	Highest Price	6.78	6.73	7.28	85.0%	9.27
	Ciprofloxacin	Lowest Price	3.55	2.40	6.79	100.0%	4.85
16	Co-trimoxazole suspension	Brand	#			5.0%	
	Co-trimoxazole suspension	Highest Price	#			0.0%	
	Co-trimoxazole suspension	Lowest Price	1.29	0.83	1.32	77.5%	0.24
17	Doxycycline	Brand				0.0%	
	Doxycycline	Highest Price	9.30	1.80	14.09	55.0%	4.86
	Doxycycline	Lowest Price	1.82	1.50	14.09	97.5%	0.95
18	Erythromycin powder for suspension	Highest Price	0.94	0.94	0.94	15.0%	0.50
	Erythromycin powder for suspension	Lowest Price	0.94	0.65	0.94	55.0%	0.50
19	Erythromycin Stearate	Highest Price	3.48	2.75	3.48	35.0%	4.81
	Erythromycin Stearate	Lowest Price	2.75	2.03	3.48	82.5%	3.81
20	Gentamicin eye drops	Brand	#			5.0%	
	Gentamicin eye drops	Highest Price	#			2.5%	
	Gentamicin eye drops	Lowest Price	0.97	0.53	1.06	65.0%	1.57
21	Gentamicin injection	Highest Price	1.92	1.76	2.15	15.0%	3.83
	Gentamicin injection	Lowest Price	1.84	1.51	1.96	57.5%	3.66
22	Norfloxacin	Highest Price	#			5.0%	
	Norfloxacin	Lowest Price	4.72	2.04	4.77	95.0%	4.85
23	Ofloxacin	Highest Price	5.22	2.74	5.70	92.5%	8.81
	Ofloxacin	Lowest Price	2.74	1.48	5.70	97.5%	4.62
24	Roxithromycin	Highest Price	*			17.5%	7.80
	Roxithromycin	Lowest Price	*			92.5%	7.80

* MSH price is not available therefore MRP cannot be calculated.

# MRP is not calculated if the antibiotic is not available at minimum of 4 outlets.

*N.A* The brand version of that particular antibiotic is not available in India.

*INR* Indian Rupees (Local currency).

#### ***Price variation between highest and lowest generic***

**Figure 1:**
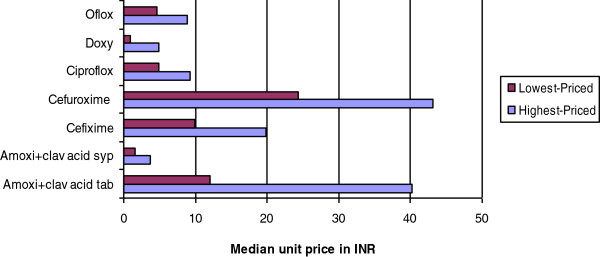
**Price variation for a few antibiotics in the highest-priced and lowest-priced generic available at private retail pharmacies.** Oflox = Ofloxacin; Doxy = Doxycycline; Ciproflox-Ciprofloxacin; Amoxi + clav = Amoxicillin + clavulanic acid; INR – Indian Rupees (Local currency).

For certain medicines huge price variation was observed for highest-priced and lowest-priced generic available at the surveyed retail facilities. The mean MPR of highest and lowest-priced generic is shown in Figure 1 and the highest variation of 5.1 times was seen for doxycycline, followed by amoxicillin + clavulanic acid tablet (3.3 times).

#### ***Overall availability***

Except for two antibiotics all other antibiotics were available in the private sector (Table 8). If only one version of the medicine (apart from originator for seven core medicines) was available then that medicine’s name and price became the lowest-priced generic available. Results were similar at chain pharmacies although the availability of certain antibiotics like cephalexin was more at chain pharmacies.

#### ***Antibiotics with very poor availability***

The two antibiotics that had very poor or no availability {0.0%-2.5% in any version (OB or generic)}, were, ampicillin suspension and benzathine penicillin powder. Both the antibiotics are included in the Delhi state EML.

#### ***Antibiotics with mainly one trade name version availability***

A few antibiotics were generally available at both retail pharmacies and chain pharmacies with only one trade name which was usually the most popular brand name. These antibiotics were: ceftriaxone injection, cefuroxime suspension, cephalexin, cephalexin syrup, co-trimoxazole suspension, erythromycin stearate, gentamicin eye drops, gentamicin injection, norfloxacin, and roxithromycin.

#### ***Price variations for few antibiotics in public and private sector***

We expect medicines to be more costly in private retail sector than the public procurement price. For few antibiotics the price variations was high as could be seen in Tables 4 and 8. It was found that median MPR of lowest-priced generic (LPG) in private sector for cefuroxime suspension and cephalexin syrup were seven times; amoxicillin500 mg, amoxicillin250 mg, cefuroxime were five times; and for cephalexin 6.5 times higher in the private retail sector as compared to median MPR for public procurement price.

### Price and availability of high-end antibiotics surveyed

#### ***Availability and price of surveyed high-end antibiotics at tertiary and secondary care facilities of public sector***

All five procurement agencies were procuring or had fixed the rates for vancomycin and meropenam injection (Table 9). For the two oral antibiotics, gemifloxacin and moxifloxacin tablets procurement price was available only for MCD though these were not available in their hospitals during the survey. Colistin injection was not procured by any agency. All five tertiary care hospitals had one or more surveyed antibiotics (Table 10). Out of the ten secondary care hospitals, two of Delhi government and one from MCD had no surveyed high-end antibiotics. The availability pattern indicates that the most common high-end antibiotic available in the hospitals is ceftazidime injection followed by meropenam and vancomycin injection.

**Table 9:** Antibiotic unit price in local currency (INR) for surveyed high-end antibiotics in public and private sector

**Antibiotic name**	**Public sector procurement price(procurement agency)**	**Private sector (median unit price at retail pharmacies)**	**Private sector (median unit price at chain pharmacies)**
Cefepime 1 g Inj	65.52 (MCD),	-	145.00
	80.22 (CG1)		
Ceftazidime 1 g vial	30.75 (CPA)	-	416.70
	32.80 (CG1)		
	37.85 (CG2)		
Colistin 1000000units/vial	-	-	-
Gemifloxacin 320 mg tab/cap	15.27 (MCD)	14.90	14.90
Imipenem + cilastin	386.98 (CG2)	-	1200.00
500 mg + 500 mg Inj	567.00 (CG1)		
Meropenem 500 mg Inj	365.54 (MSO) 1 g inj	1347.00	1347.00
	1083.00 (MCD) 1 g inj		
	1606.50 (CG2) 1 g inj		
	1662.20 (CG1)		
	850.50 (CPA) 500 mg;		
	1606.00 1 g inj		
Moxifloxacin 400 mg tab/cap	29.60 (MCD)	69.55	69.55 & 83.60
Vancomycin 500 mg inj	56.70 (MSO)	-	-
	58.80 (CPA & CG1)		
	64.67 (MCD)		
	65.45 (CG2)		

*INR* Indian Rupees (Local currency).

**Table 10:** Availability of surveyed high-end antibiotics at secondary care and tertiary care public facilities and at private sector facilities

**Antibiotic name**	**Public sector (n = 15)**	**Retail pharmacies**	**Chain pharmacies**
		**(n = 40)**	**(n = 40)**
Cefepime 1 g Inj	20.0%	0.0%	10.0%
Ceftazidime 1 g vial	73.3%	5.0%	20.0%
Colistin 1000000units/vial	0.0%	0.0%	2.5%
Gemifloxacin 320 mg tab/cap	0.0%	37.5%	50.0%
Imipenem + cilastin500mg + 500 mg Inj	13.3%	0.0%	22.5%
Meropenem 500 mg Inj	60.0%	32.5%	52.5%
Moxifloxacin 400 mg tab/cap	0.0%	40.0%	72.5%
Vancomycin 500 mg inj	53.3%	5.0%	7.5%

##### Availability and price of surveyed high-end antibiotics in private sector

At private retail pharmacies the availability of high-end antibiotics was poor (Table 10). Still meropenam injection was available at 13 pharmacies and gemifloxacin and moxiflocin tablets were available at 15 and 16 pharmacies out of 40 surveyed retail pharmacies. Compared to retail pharmacies, availability of high-end antibiotics was better at chain pharmacies moxifloxacin, gemifloxacin, and meropenam injection being available at 72.5%, 50.0%, and 52.5% of these pharmacies. Prices of these antibiotics were similar to the prices available at retail pharmacies, except that two versions (trade names) of moxifloxacin were available at five chain pharmacies.

## Discussion

This survey has provided a snap shot of availability of various antibiotics in the community in Delhi in both the public and private sectors. The methodology used for the survey is a standardized methodology for measuring access to essential medicines. Results from these WHO/HAI medicine price surveys are used for situation analysis and to plan interventions to improve access to essential medicines for global population [19-21].

Despite the strengths, the WHO/HAI methodology has a few limitations. First, availability and price are determined for a specific list of survey medicines, and do not account for alternate dosage forms of these medicines or therapeutic alternates. Availability data only refer to the day of data collection at each facility and might not indicate average availability of medicines over time. However, since the survey was done in several facilities over a period of time (2–3 months), the data provide a reasonable estimate of the overall situation and are indicative of the real-life situation faced by the patients on a daily basis. For this survey, data from 68 public primary care and 10 public secondary care facilities, and 80 private pharmacies was collected over a period of three months. It is only for an individual tertiary care facility (n = 5) that the availability reported is for one particular day only. The list of antibiotics surveyed for the present survey included those commonly used in both public and private sector as determined from our previous work on trends of antibiotic used in the community in Delhi [11] and also the strength and dosage form as mentioned in the Delhi state EML [18].

It is generally agreed that rapidly increasing antimicrobial resistance is due to inappropriate use of antibiotics. There are many forms of inappropriate use, for example unnecessary use of antibiotics, failure to prescribe ac cording to guidelines or non-availability of standard treatment guidelines, non-availability of appropriate antibiotics, availability of antibiotics without prescription, inappropriate self-medication and non-adherence to prescribed dosing schedules by patients [22]. We found in this survey that many newer antibiotics which were not in the list of Delhi state EML for primary care were available at primary care facilities of both Delhi government (GNCT, Delhi) and MCD run facilities. The survey revealed that all antibiotics on the list of Delhi state EML, even those were supposed to be reserve antibiotics for hospitals like cefuroxime, were available in dispensaries (Primary care facilities) of Delhi government and MCD. Three antibiotics surveyed, namely, amoxicillin + clavulanic acid tablet and syrup and cefixime, were not in the Delhi state EML of 2007 (used at the time of the survey) and we did not get procurement price for them from CPA-GNCT, Delhi. However, they were all available at primary care and secondary care facilities of GNCT, Delhi and MCD. Although, the revised Delhi state EML of 2010 [23] has these three antibiotics, procurement at the time of the survey did not use this list and it would seem that these antibiotics were procured following some other mechanism. Our earlier surveys conducted in GNCT, Delhi primary care also found that newer antibiotics meant for hospital use were available at primary care and were frequently used by the doctors [11,12,24]. The present survey also revealed that all antibiotics on the Delhi state EML for hospitals, even those for in-patients, were also available in the dispensaries (primary care). One procurement officer in CPA department of GNCT Delhi stated that all procured antibiotics are distributed to the dispensaries and that the Delhi state EML for dispensaries is not used for distribution. Unfortunately MCD does not have an EML and no separate lists for primary care, secondary care and tertiary care facilities.

Unnecessary or inappropriate use of antibiotics must be curbed immediately. Inappropriate availability will lead to inappropriate use. The next step is to have the national or local antibiotic policy including the development of an appropriate EML by level of facility, and monitoring to ensure compliance of procurement and supply with the EML [25]. Antibiotics should be available for use at the public sector according to standard treatment guidelines and the essential medicines list. Findings of this survey clearly reveal that though the Delhi state government (GNCT, Delhi) did have an EML, it was not followed for procurement and distribution to primary care. The other two agencies, MCD and facilities of central government had a procurement list which differed from the Delhi state EML and which may not have been developed according to patient need. Unlike a procurement list, development of an EML requires a committee of expert(s) who clearly understand the concept and importance of essential medicines and will prepare and revise the EML including antibiotics on the basis of essential medicine concepts [26]. Therefore, it is recommended that all providers should follow the essential medicine concept and that the EML be adapted to the differing medicines needs in primary care and tertiary care health care.

It is important to implement the EML and ensure that procurement and distribution of medicines (antibiotics) follow the EML according to various level of health care. None of the antibiotics which were meant for primary care (dispensaries) had 100% availability or were available at all the surveyed facilities. Two antibiotics, amoxicillin500 mg and norfloxacin were available between 80.0%-85.0%; for children amoxicillin250 and co-trimoxazole suspension was available at 71.9% and 56.3% facilities. Variety of antibiotics was available not according to the STGs or EML but according to the supply in the central store. Availability of antibiotics in secondary care hospitals was better than at tertiary care facilities. This finding indicates probably that smaller hospitals are managing their store and quantification better than the tertiary care hospitals in GNCT, Delhi.

Compared to GNCT-run facilities, access to antibiotics in MCD-run facilities was poor, none of the surveyed antibiotics having more than 80% availability at surveyed primary care facilities and only 4 antibiotics (ciprofloxacin, doxycycline, amoxicillin + clavulanic acid tablet and syrup) being available in 50% of facilities. While some essential antibiotics which should be available at primary care were poorly available, other second generation antibiotics like ofloxacin, cefuroxime, and cefixime had better availability than first generation antibiotics at primary care facilities. Availability of antibiotics at tertiary care facility was also poor and the only antibiotic available for children was amoxicillin + clavulanic acid syrup.

Findings of the survey provide a good baseline and evidence to administrators, managers, and policy makers to prepare and put in action essential medicine list and a good antibiotic policy [27]. A committee on EML and antibiotic policy should ensure that antibiotics are selected, procured, distributed and used more critically than has been the case up to the present time at different level of health care facilities.

For the public sector procurement price of surveyed antibiotics, it was strange to find that the older off-patented antibiotics viz., amoxicillin dosage forms and strength, ampicillin suspension, doxycycline and erythromycin had higher MPR than most of the newer antibiotics. This indicates the Indian manufacturers are selling off-patented antibiotics at a comparatively higher price than newer antibiotics. Variation in procurement price for different agencies was found and for quite a few of antibiotics, the procurement price of central government tertiary care facility doing independent procurement was much higher than other agencies. These findings confirm that pooled procurement decreases the procurement price [28,29] and local purchases of medicines done by individual facility cost higher [30].

At the private sector retail pharmacies, few antibiotics, like ampicillin suspension and benzathine penicillin powder for injection were either not available or available at one or two pharmacies only. This indicates that these antibiotics are generally not prescribed by doctors. Newer members from various classes, which are more expensive than the first generation of antibiotics (e.g. amoxicillin + clavulanic acid tablet and syrup, cefixime, cefuroxime, azithromycin, ofloxacin, roxithromycin), were always available at private pharmacies, as found in our previous surveys [11]. For a few antibiotics, only one trade name that is more costly (trade name or branded product) product was available.

These findings indicate that doctors are prescribing mainly the branded medicines that are pushed by the companies through their representatives and that pharmacists will stock those medicines for which they usually get prescriptions. In India generic substitution is legally not allowed. The brand name (trade name) written by a doctor can not be substituted with another. Some countries do have generic prescribing policies and/or generic substitution and such generic policies would be very efficient but it requires a high level of commitment from government and regulators. Excellent availability of antibiotics in the private retail pharmacies and chain pharmacies may be good news for patients from an access viewpoint. However, in India, like other developing countries, antibiotics may be obtained easily from private retail pharmacies without a prescription [31], contrary to the Indian government regulation and drug schedules. Indian pharmaceutical GMP guidelines are given in Drugs and Cosmetics Act 1940. Rules are given for pharmaceuticals and schedules are there to comply with the rules. “Schedule H” is a class of prescription drugs in India under Drugs and Cosmetics rules. Antibiotics come under ‘Schedule H’ and legally should not be dispensed without a prescription.

However, pharmacists themselves prescribe and dispense antibiotics usually for one or two days for conditions like acute diarrhea, sore throat etc. and follows the prescription of neighborhood practicing doctors [32]. Ministry of Health and Family Welfare, Government of India has recently prepared National Policy for Containment of Antimicrobial Resistance-India that covers a range of actions to be taken including introducing a separate schedule H1 for the sale of antibiotics. Under this schedule H1 third generation antibiotics and all newer antibiotic sales will be restricted [33]. However, this has yet to be implemented.

In the private sector availability of newer respiratory fluoroquinolones, gemifloxacin and moxifloxacin was good. Even meropenam injection was available at 32.5% retail pharmacies. Availability of these high-end antibiotics was better at retail chain pharmacies. Surprisingly, the retail pharmacies including the chain pharmacies where these antibiotics were available were not near the big hospitals but rather in the peri-urban areas or not so posh areas of Delhi.

## Conclusion

Availability of some essential antibiotics is sub-optimal while other non-essential antibiotics are freely available in both the public and private sectors. Only one public sector agency had an EML which was only partially followed in procurement and distribution. Some newer reserve antibiotics are freely available in the private sector and priced lower than the older essential non-reserve antibiotics. Such a situation is likely to lead to inappropriate use of antibiotics and contribute to AMR. It is urgent that managerial and regulatory interventions be initiated to ensure that all public sector procurement follows an EML that has been developed in an evidence-based manner and that newer antibiotics are not available OTC in the private sector at inappropriately low prices.

## Competing interests

All authors declare that they have no competing interests.
